# Sleep assessment in adults with Down syndrome: correlation between functionality and polysomnographic findings

**DOI:** 10.1055/s-0043-1768670

**Published:** 2023-06-28

**Authors:** Marilia Rezende Callegari, Kelly Brito dos Santos, Barbara Valente de Oliveira, Ana Rita Avelino Amorim, Raquel Cymrot, Silvana Maria Blascovi-Assis

**Affiliations:** 1 Universidade Presbiteriana Mackenzie, Centro de Ciências Biológicas e da Saúde, São Paulo SP, Brazil.

**Keywords:** Down Syndrome, Sleep, Sleep Wake Disorders, Polysomnography, Physical Functional Performance, Síndrome de Down, Sono, Transtornos do Sono-Vigília, Polissonografia, Desempenho Físico Funcional

## Abstract

**Background** Sleep disorders have a negative impact on health, being associated with neurocognitive problems, cardiovascular diseases and obesity, influencing children's development and learning.

**Objective** To assess the sleep pattern of people with Down syndrome (DS) and correlate changes with functionality and behavior.

**Methods** A cross-sectional study was conducted to evaluate the sleep pattern in adults with DS > 18 years old. Twenty-two participants were assessed using the Pittsburgh Sleep Quality Index, the Functional Independence Measure and the Strengths and Difficulties Questionnaire, and the 11 who presented indications of disorders by the screening questionnaires were referred to polysomnography. Statistical tests were performed using a significance level of 5%, including sample normality tests and correlation tests (sleep and functionality).

**Results** Impairment in sleep architecture was found due to an increase in the rate of awakenings in 100% of the participants, a decrease in the number of slow waves, and a high prevalence of sleep disordered breathing (SDB), with higher averages in the Apnea and Hypopnea Index (AHI) in the group. There was a negative correlation between sleep quality and global functionality ( *p* = 0.011) and the motor ( *p* = 0.074), cognitive ( *p* = 0.010), and personal care ( *p* = 0.072) dimensions in the group. Global and hyperactivity behavior changes were related to worse sleep quality ( *p* = 0.072; *p* = 0.015, respectively).

**Conclusion** There is an impairment in the sleep quality of adults with DS, with an increase in the rate of awakenings, a decrease in the number of slow waves, and a high prevalence of SDB affecting this population in the functional and behavioral aspects.

## Resumo

**Antecedentes** Os distúrbios de sono têm impacto negativo na saúde, estando associados a problemas neurocognitivos, doenças cardiovasculares e obesidade, influenciando no desenvolvimento e aprendizado.

**Objetivo** Avaliar o padrão de sono de pessoas com síndrome de Down (SD) e correlacionar as alterações com a funcionalidade e comportamento.

**Métodos** Foi realizado um estudo transversal para avaliação do padrão de sono em adultos com SD > 18 anos. Foram avaliados 22 participantes, através do Índice de Qualidade do Sono de Pittsburgh, da Medida de Independência Funcional e do Questionaário de Capacidades e Dificuldades. Os 11 participantes que apresentaram indicativos de presença de distúrbios pelos questionários de triagem foram indicados a polissonografia. Os testes estatísticos foram realizados com nível de significância de 5%, incluindo testes de normalidade e testes de correlação (sono e funcionalidade).

**Resultados** Foi encontrado prejuízo na arquitetura de sono pelo aumento do índice de despertares em 100% dos participantes, diminuição na quantidade de ondas lentas, e alta prevalência de distúrbio respiratório do sono (DRS), com maiores médias nos Índices de Apneia e Hipopneia (IAH). Houve correlação negativa entre a qualidade de sono e a funcionalidade global ( *p* = 0,011), e as dimensões motora ( *p* = 0,074), cognitiva ( *p* = 0,010) e cuidados pessoais ( *p* = 0,072). As alterações de comportamento global e comportamentos de hiperatividade foram relacionados à pior qualidade do sono ( *p* = 0,072; *p* = 0,015, respectivamente).

**Conclusão** Existe prejuízo na qualidade de sono de adultos com SD, com aumento de índice de despertares, diminuição na quantidade de ondas lentas, e alta prevalência de DRS, afetando essa população nos aspectos funcionais e comportamentais.

## INTRODUCTION

Down syndrome (DS), also known as trisomy 21 (T21), presents several clinical characteristics of DS, such as hypotonia, micrognathia, decreased pharynx diameter due to mandibular hypoplasia, decreased airway diameter, and flaccid supraglottis, have the potential to raise the risk of sleep disorders because such features can impair breathing at this phase, increasing the risk mainly of sleep-disordered breathing (SDB). Adenoid and tonsil hypertrophy, associated with decreased pharyngeal muscle tone and overweight, further contribute to this disorder. 1 2 3 4 5

The most significant risk of sleep disorders in this population may include difficulty initiating or maintaining sleep, excessive daytime sleepiness and obstructive sleep apnea (OSA). Santos et al. 6 indicate that the primary sleep disorder associated with the DS population is OSA. Compared with typical children, DS people have a 74% prevalence of sleep disorders when assessed by screening questionnaires, compared with 36% for the typical population. The same study also demonstrates 57.1% of OSA in children with DS set by polysomnography (PSG) test. 4

Sleep problems will persist into adolescence, except for parasomnias, more prevalent in younger children and that decrease with age. Even with an adverse history of symptoms such as snoring and breathing pauses, polysomnographic evaluations are recommended early (< 5 years) as the high prevalence is found in all age groups. 4 7 8

Studies showed that sleep disorders in DS are negatively associated with performing daily activities; that is, children who have higher scores on the sleep disturbance scales have worse performance on the scales of habits and activities of daily living. 9

Few studies address the adult population with DS. The study by Giménez et al. 10 evaluated the prevalence of sleep disorder (SD) in adults with DS using methodological strategies of self-report (questionnaires) associated with objective measures (PSG), finding a high prevalence of severe sleep disorders and OSA. Besides, self-reported sleep by questionnaires does not reflect sleep disorders in this population and is not perceived by caregivers, suggesting that the presence of cognitive impairment masks these data. The PSG results also suggest a premature process of changes in sleep architecture in DS, with early changes in patterns compared with typical groups. They concluded that OSA screening should be routinely recommended for this population because treating SD can contribute to healthy aging. 10

Early detection of sleep disorders in people with intellectual disability (ID) is essential and should be investigated for their adverse effects on development and may be among the predictors of cognitive decline in adulthood. Therefore, it is vital to understand the architecture and the main changes in sleep in people with DS to design preventive or supportive interventions that maximize the benefits of therapies related to the SD and other therapies.

Hence, the present study aimed to evaluate the sleep pattern of adults with DS and to correlate changes with functionality and behavior.

## METHODS

The present cross-sectional, descriptive, and correlational study was used to assess sleep patterns in adults with DS in a nonprobabilistic convenience sample selected by the researchers. The Research Ethics Committee of Universidade Presbiteriana Mackenzie (UPM, in the Portuguese acronym) approved the study under Opinion No. 3.450.082.

Individuals with a confirmed diagnosis of DS > 18 years old were included. Those who had associated comorbidities such as autism spectrum disorder, transient or permanent neurological or orthopedic injuries and those who did not complete the questionnaire in the first phase were excluded.

In the first phase, the following instruments were used: Pittsburgh Sleep Quality Index for initial screening of sleep problems 11 ; Functional Independence Measure (FIM) for assessment of functional performance 12 ; Strengths and Difficulties Questionnaire (SDQ) for tracking behavioral changes and emotional and social characteristics, 13 all of which were answered by their parents or guardians. Even though the age range indicated (2 to 17 years) by the authors of the SDQ, it has already been used and recommended in adults with DS in the study by Glenn et al. 14

In the second phase, participants were screened using questionnaires to perform the PSG test. Polysomnography is the standard gold test to quantify, qualify and document sleep through multiple physiological parameters such as electroencephalogram (EEG), electromyogram (EMG), electrocardiogram (ECG), electrooculogram (EOG), oronasal airflow, respiratory effort, and pulse oximetry, providing information on sleep architecture and the presence of different types of disorders.

The tests were performed according to international guidelines, at the level I of specificity, that is, using at least seven channels including EEG, EOG, submental EMG, ECG, oronasal airflow, respiratory movement and peripheral oxyhemoglobin saturation; body position documented or objectively measured by position sensors; EMG leg movement; and constant supervision of the examination by a technician. 15 16

Those who showed signs of disorders by the screening questionnaires were indicated for the examination. Those who presented some comorbidity that compromised the polysomnography data, such as seizures or untreated acute infection, were discontinued in the second phase.

The variables of the polysomnography analysis were used sleep efficiency, abnormal sleep architecture due to the decrease or absence of sleep stages, presence of sleep disturbance due to an increase in the number of micro-arousals (> 10/hour), and presence of sleep disordered breathing due to AHI (> 5/hour), SatO2 < 85%, and the presence of snoring.

At the end of the collections, all received a detailed report with the sleep and performance assessment results, a letter of guidance and care regarding sleep problems, and a copy of the PSG exam. Those who had confirmed the diagnosis of some sleep disorder were referred to a specialized team linked to the Brazilian Unified Health System for treatment.

### Data analysis

Statistical tests were performed using the MiniTab software, including sample normality tests; correlation tests – sleep and functionality/performance, using a significance level of 5%.

The magnitude of the correlations obtained was analyzed based on Cohen parameters, 17 defined as: small (0.10 < r < 0.29); medium (0.30 < r < 0.49) and large (0.50 < r < 1.00). In this correlation analysis, *p* ≤ 0.05 was adopted as statistically significant, and *p* ≤ 0.10 as marginally significant.

## RESULTS

In the first phase of the study, 25 people between 18 and 34 years old participated, averaging 23.87 (± 4.78) years old. Two participants were excluded, one for unconfirmed diagnosis and one for incomplete fulfillment of the questionnaires. So, the study continued with 23 participants, 12 males and 11 females.

Analyzing the questionnaires completed during the interviews, it was observed that 18 participants (78.2%) achieved scores indicating sleep problems displayed for PSG. Despite the number of participants indicated for the exam, only 11 families agreed to undergo the PSG test.

One more participant was excluded from the statistical analyses due to data discrepancy (outliers). As a result, the data of 22 participants were analyzed. Descriptive statistics ( Table 1 ) were performed for later correlation calculations among Pittsburg variables and the three domains of the FIM Scale.

**Table 1:** Descriptive statistics for the Pittsburgh scale, FIM, SDQ ( *n* = 22)

Variable	Average	SD	CV	Minimum	Maximum
Age	24.09	4.78	19.84	18	34
Pittsburgh	6.13	3.35	54.70	2	16
Total FIM	113, 18	11.25	9.94	91	126
Motor dimension	86.86	4.91	5.65	77	91
Cognitive dimension	26.32	7.57	28.76	12	35
Personal care	38.36	4.28	11.16	31	42
Total SDQ	14.59	7.66	52.48	0	32
Emotional symptoms	4.45	2.65	58.30	0	5
Behavioral problems	2.81	2.28	80.94	0	10
Hyperactivity	3.81	3.12	81.88	0	10
Relationship problems	3.409	2.19	64.43	0	8
Prosocial behavior	7.50	2.55	34.12	1.0	10

Abbreviations: CV, coefficients of variation; FIM, Functional Independence Measure; SD, standard deviation; SDQ, Strengths and Difficulties Questionnaire.

Negative linear correlations were found by the Pearson test ( Table 2 ), considered of great magnitude between the FIM Scale and the Pittsburgh Scale (r = - 0.531; *p* =0.011), between the cognitive dimension of FIM and Pittsburg (r = - 0.538; p = 0.010), and the locomotion domain (r = - 0.625; *p* = 0.002). And of medium magnitude between Pittsburg and the motor dimension (r = - 0.389; *p* = 0.074), the personal care domain (r = - 0.391; *p* = 0.072), and communication (r = - 0.417; *p* = 0.054). The *p-* values of the negative linear correlations were significant for two of the analyzes at the 5% level and marginally significant for 2, indicating that, for adults, the fewer sleep problems, the better the functional performance.

**Table 2:** Linear correlations between the Pittsburgh case versus FIM and SDQ

Functionality/behavior	Pittsburgh	
	r	*p-* value
Total FIM	−0.531	0.011*
Motor FIM	−0.389	0.074
Cognitive FIM	−0.538	0.010*
Personal care FIM	−0.391	0.072
Sphincter control FIM	0.055	0.072
Locomotion FIM	−0.625	0.002*
Communication FIM	−0.417	0.054
Total SDQ	0.391	0.072
Hyperactivity	0.511	0.015

Abbreviations: FIM, Functional Independence Measure; SDQ, Strengths and Difficulties Questionnaire. Note: * *p* ≤ 0.05.

The SDQ score values punctuate the degree of difficulties presented and are interpreted as normal (0–13), borderline (14–16), and abnormal (17–40) in the total score and the subscales of emotional symptoms, behavioral problems, hyperactivity, relationship problems, prosocial behavior. In this behavior analysis, 11 were considered normal, 3 borderline, and 8 abnormal.

A marginally significant average magnitude correlation was found between the Pittsburgh and the SDQ total score (r = 0.391; *p* = 0.072). There was also a high magnitude correlation between the Pittsburgh total index and hyperactivity behavior (r = 0.511; *p* = 0.015). There was no significant correlation between the sleep scale and the other SDQ subscores.

### Analysis of polysomnography tests

In the PSG examination phase, from the 18 patients indicated for the test, 3 were excluded because of seizures, 4 refused to undergo the examination, and 11 underwent PSG.

In the descriptive evaluation, 81% (9 participants) had decreased or very decreased sleep efficiency, and 100% of those evaluated had abnormal sleep architecture, being reduced or absent slow-wave sleep, and REM sleep, the characteristics of this population. The descriptive data of these variables are detailed in Table 3 . Only one participant had no respiratory disorder. Three participants had mild respiratory disorder (RD) while three others had moderate, two severe, and two very severe.

**Table 3:** Descriptive statistics ( *n* = 11)

Variable	Average	SD	CV	Minimum	Median	Maximum
Age	23.18	3.40	14.67	19.00	23.00	31.00
Sleep efficiency (%)	65.79	20.18	30.67	35.70	66.60	92.60
Micro-arousal (index/time)	35.85	17.74	49.49	14.10	33.70	72.80
AHI/h	32.68	30.16	92.29	1.40	22.60	102.30

Abbreviations: AHI, apnea-hypopnea index; CV, coefficients of variation; SD, standard deviation.

There was no significant correlation between the AHI, the FIM Scale, and the dimension of FIM, and between AHI, SDQ and the other SDQ subscores.

## DISCUSSION

The literature points out that the lack of adequate sleep, both in quality and in adequate amounts, can lead to adverse health outcomes with daytime consequences such as impaired function, fatigue, and memory changes, impacting development and contributing to biological and cognitive aging, as well as the onset of chronic diseases. 18 19

The present study is a pioneer in the Brazilian population with DS using PSG level 1 for the assessment. It provided data on the quality of the population and the most major sleep disorders, formed by a volunteer group of adults, contributing to a better evaluation and treatment of this public.

The results corroborate the study by Breslin et al. 7 about children and adolescents with DS (from 7 to 18 years old), which indicated that sleep problems in this population are not restricted to RDs, as well as the study by Trois et al. 20 who demonstrated a decrease or absence of slow-wave sleep, with an increased rate of arousals in DS adult subjects.

In the study, we observed a 78% rate of alterations in sleep quality in the assessments using questionnaires (PSQI > 5), which is similar to some studies in the adult population, such as 61% identified in the study by Carvalho et al., 21 and in previous studies according to the meta-analysis conducted by Ridore et al. 22 This high rate of sleep problems in the studied group can be explained by the composition, sample selection, and voluntary participation in the study, and the interest of family members who were possibly already concerned and attentive to the topic. However, the low adherence to the PSG test, considering that 18 were preselected and only 11 underwent the test, shows the difficulty of monitoring this population, being a limitation to be considered in the results presented here.

According to Stores, 23 there are several reasons for the limited number of studies on sleep in adults with DS, like the difficulties in carrying out the assessments and the little information reported on sleep disorders in the International Classification of Sleep Disorders.

Changes in sleep architecture found in DS people may increase the factors that indicate early aging in this population, as described in the literature, 24 including sleep parameters as another factor to be evaluated.

Sleep efficiency < 85%, that is, decreased concerning normal values, demonstrated by the present study as significantly lower, has already been previously reported by the study by Trois et al. 20 in this population, with a decrease in normality values of 69%, and according to the same study, this worsening of sleep efficiency was not related to the atypical night of the test.

The assessed population presented significant sleep fragmentation, with arousals rates well above the normal values (normal < 10/hour), with an average of 33.15/hour. Sleep fragmentation in healthy adults has been associated with excessive daytime sleepiness, impaired reaction time, and deficits in executive function. In adults with DS, a factor that explains the increased prevalence of dementia in this population may be related to hypoxemia. 20

Other important factors associated with sleep fragmentation and SDB in DS are cardiovascular complications, which can be even more dangerous in individuals with DS. There may be residual sequelae of congenital cardiomyopathy, and untreated Obstructive Sleep Apnea Syndrome (OSAS) can contribute to early mortality. 20

According to the Sleep Disorders Manual, the diagnosis of SDB in adults requires a frequency of obstructive respiratory events more significant than 15/hour, or > 5 to 15 events/hour associated with signs/symptoms. 25 Following the mentioned reference values, 100% presented snoring, only 1 participant (10%) had no respiratory disorder, and the others had AHI ranging from mild to very severe. None of the studies found in the literature on the population with DS refer to snoring but emphasize SDB and OSA research.

Regarding the severity of OSAS, the data found in the assessed group suggest a greater severity when compared with normative data in the literature of the typical Brazilian population (16.9%). 26 However, the rate of participants with moderate to very severe conditions (64%) corroborates the prevalence of 81.6% identified in a similar study in the Brazilian population with SD. 21 In addition, 72% of adult participants achieved an O2 saturation Nadir (minimum oxygen saturation during the sleep study) < 85%, compared to 8% of a typical control population in the literature. 27

According to Capone et al *.* , 28 OSAS is a common comorbidity in adolescents and adults with DS and depression. Recognizing this association is essential for understanding the disease and managing mood disorders and functional decline adequately. The literature highlights the increased prevalence of dementia and Alzheimer's disease in adults with DS, which in theory may be related to the hypoxemia and sleep fragmentation associated with individuals with OSAS. 20

The data from the present study also corroborate other research described in the literature, which state that sleep problems negatively impact health in association with neurocognitive issues, mood, anxiety, attention, and hyperactivity disorders. 4 Also, sleep disorders are negatively associated with the performance of daily activities in children with DS, 9 in agreement with the data found here for the adult population.

In the evaluation of functionality in the motor, cognitive, locomotion and personal care dimensions, a significant negative linear correlation was found, indicating that the higher the scores on the sleep problems scale, the worse the scores on the functionality scale, observing the impact of the sleep disorder on functionality. The behavior assessment also showed high-magnitude correlations between the sleep scale and hyperactivity behaviors, including restlessness, distraction, and inattention.

Some variables that can be considered in the analysis of sleep disorders were not considered in the present study, such as the presence of comorbidities associated with DS, such as thyroid dysfunction, corrected cardiopathies, and other secondary comorbidities, being limitations of the study and suggestions for future research. However, it is worth mentioning that, in the study conducted by Trois et al., 20 no relation was found between hypothyroidism and the severity of obstructive apnea.

Al-Sharman et al. 29 demonstrated that sleep improves the learning of a functional motor skill, indicating that an emphasis should be given to treating sleep disorders and ensuring adequate sleep for individuals with compromised neurological conditions or who participate in physical rehabilitation. And as verified by the present study, in agreement with the literature, these disorders are often underdiagnosed and deserve special attention in the investigation of this population.

Based on the findings of the present study, it was possible to identify in the studied sample some sleep alterations that corroborate the international literature. The PSG test showed impairment in sleep architecture due to an increase in the rate of arousals, a decrease in the number of slow-wave sleep in adults, and a high prevalence of SDB.

Polysomnographic findings confirmed the assessment through questionnaires. However, only PSG is sensitive to determine the severity of sleep disturbances, quantified by recording sleep fragmentation, respiratory disturbance, and changes in architecture.

There was a negative linear correlation between the sleep assessment and the motor, cognitive, locomotion and personal care dimensions of adults with DS. Alterations in global behavior and hyperactivity behaviors were related to worse sleep quality.

It is expected from the present study to follow up with future works whose concern is focused on sleep issues and other clinical and functional implications of people with DS since sleep disorders when, left untreated, can cause functional impairments, implying barriers to social inclusion.
